# Exercise for optimizing bone health after hormone-induced increases in bone stiffness

**DOI:** 10.3389/fendo.2023.1219454

**Published:** 2023-09-18

**Authors:** Julie M. Hughes, Katelyn I. Guerriere, Kristin L. Popp, Colleen M. Castellani, Stefan M. Pasiakos

**Affiliations:** ^1^ Military Performance Division, United States Army Research Institute of Environmental Medicine, Natick, MA, United States; ^2^ Oak Ridge Institute for Science and Education (ORISE), Oak Ridge, TN, United States

**Keywords:** endocrine, mechanobiology, mechanostat, hormones, growth, osteoanabolic therapy

## Abstract

Hormones and mechanical loading co-regulate bone throughout the lifespan. In this review, we posit that times of increased hormonal influence on bone provide opportunities for exercise to optimize bone strength and prevent fragility. Examples include endogenous secretion of growth hormones and sex steroids that modulate adolescent growth and exogenous administration of osteoanabolic drugs like teriparatide, which increase bone stiffness, or its resistance to external forces. We review evidence that after bone stiffness is increased due to hormonal stimuli, mechanoadaptive processes follow. Specifically, exercise provides the mechanical stimulus necessary to offset adaptive bone resorption or promote adaptive bone formation. The collective effects of both decreased bone resorption and increased bone formation optimize bone strength during youth and preserve it later in life. These theoretical constructs provide physiologic foundations for promoting exercise throughout life.

## 1 Introduction

In an editorial published in 2002, Dr. Ego Seeman wrote, “Each region of the axial and appendicular skeleton, each point on the external and internal surfaces along a bone’s length and circumference, is fashioned by genetic factors, local mechanical and hormonal factors, into a structure adapted to loading (1).” Of these factors, mechanical and hormonal factors are always in flux, and their interconnectedness in bone functional adaptation throughout life can reveal practical solutions for promoting bone strength and preventing bone fragility. In this paper, we review a series of well-established observations of hormonal effects on bone throughout life and interpret these within the context of synergistic and beneficial interactions with mechanical regulation of bone by exercise.

Classic examples of hormonal effects on bone that we discuss include bone growth from anabolic sex hormone secretion during puberty and subsequent functional adaptation of bone that helps explain sex-based differences in bone properties during adulthood. Contemporary examples include bone resorption that follows exogenous osteoanabolic hormone treatment for osteoporosis. In each of these scenarios, we use our working model of bone functional adaptation (2) to help decipher the synergistic physiological pathways by which hormones and exercise co-regulate bone and the modulating effects that exercise has on these processes.

We focus the review on physiologic responses of bone to hormone-induced increases in bone stiffness, which is a mechanical property that reflects the ability of bone to resist external forces without permanent changes to its structure. We begin by reviewing hormone-induced increases in bone stiffness and then discuss the role exercise serves in offsetting bone resorption or promoting bone formation. We conclude by discussing practical implications of exercise for maintaining and promoting bone health, concurrent with hormone-induced increases in bone stiffness throughout life.

## 2 Mechanical and hormonal co-regulation of bone stiffness

Beneficial effects of exercise on bone mechanical properties have long been recognized (3), as is the co-regulation of bone by hormones and mechanical loading (4, 5). Recently, we posited that bone stiffness, rather than the traditionally referenced mass or strength, is the mechanical property that governs bone adaptation to mechanical loading (2). Classic studies revealed a narrow homeostatic range of 2,000-3,000 microstrain (6, 7) in loaded bones is maintained across many different species during peak functional activities. Therefore, it can be argued that stiffness, which is the ability of bone to resist applied loads and return to its original form, is regulated by bone functional adaptation, and not mass (quantity of bone) or strength (ultimate force at which the bone breaks). The corollary is that when bone stiffness is altered for non-mechanical reasons such as hormonal stimulation, the new bone stiffness must be interpreted within the context of the prevailing mechanical environment, and as a result, bone functional adaptation may be stimulated.

To decipher the complex interactions between mechanical and hormonal interactions in the regulation of bone stiffness, and the critical role that exercise serves in these processes, we first begin with a physiologic guide for interpreting bone mechanoadaptation after increased or decreased mechanical loading. We then layer on the interaction of hormone-driven changes to the mechanical environment. This tiered approach can help provide a physiological foundation from which the significance of exercise for promoting bone health during times of hormonal and mechanical interactions can be appreciated.

### 2.1 Mechanical regulation of bone stiffness

**Figure 1:**
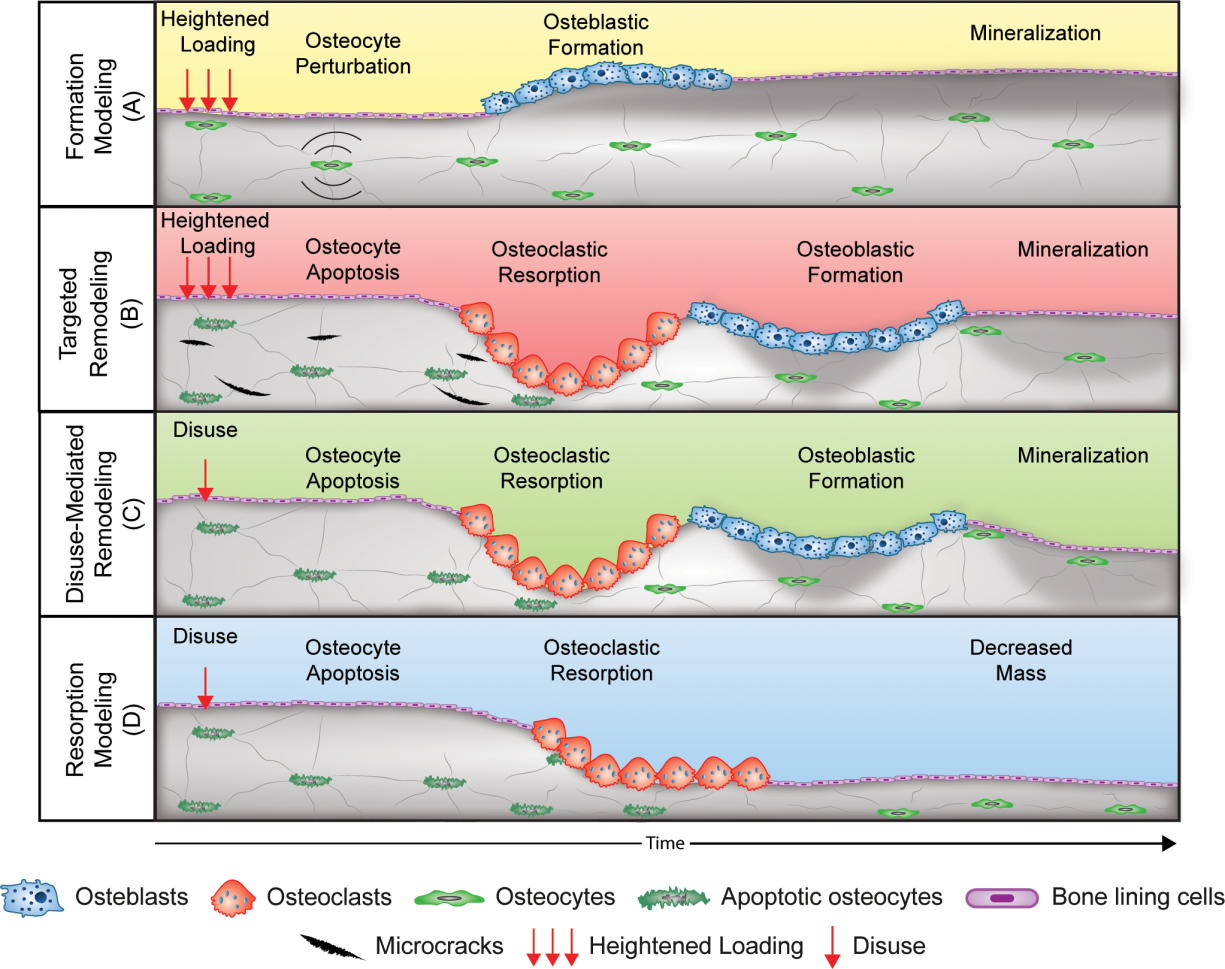
Four pathways of bone functional adaptation: Schematic of the four mechanoadaptive responses at the cellular level. Formation modeling **(A)**: osteocyte perturbation by mechanical loading induces osteoblastic bone formation on a surface. Targeted remodeling **(B)**: microcracks generated during loading stimulate osteocyte apoptosis and targeted removal of bone by osteoclasts and subsequent formation of bone by osteoblasts. Disuse-mediated remodeling **(C)**: osteocyte apoptosis with disuse stimulates bone resorption and coupled formation. Also depicted is the negative bone balance within each remodeling unit that can accompany disuse-mediated remodeling. Resorption modeling **(D)**: disuse-mediated osteocyte apoptosis stimulates osteoclastic bone resorption on a surface. Reprinted with permission from Hughes et al., Exercise and Sport Sciences Reviews, 2020 (2).

We recently proposed a theoretical model for mechanical regulation of bone stiffness (2), based on classic models (3, 8–10), that provides a guide for deciphering tissue-level responses of bone to exercise and also disuse. This model is akin to the classic model of bone functional adaptation proposed by Dr. Harold Frost, known as the Mechanostat. Our model differs in its focus on regulating bone stiffness instead of mass or strength and in recognition of four distinct mechanoadaptive pathways (**Figures 1A–D**) consisting of bone modeling (the independent action of osteoclasts or osteoblasts) or remodeling (the couple actions of osteoclasts and osteoblasts in a remodeling unit) acting in a negative feedback system. Briefly, when mechanical loading (**Figure 2.1**) on a bone of a given stiffness (**Figure 2.2**) is greater than customary (up until the point of structural failure), the bone matrix experiences strain (**Figure 2.3**) that initiates both formation modeling (**Figures 1A**, **2.4**) and targeted remodeling (**Figures 1B**, **2.5**). However, when mechanical loading is less than the bone is accustomed to, lower strain stimuli can initiate disuse-mediated bone remodeling (**Figures 1C**, **2.6**) and resorption modeling (**Figures 1D**, **2.7**).

**Figure 2:**
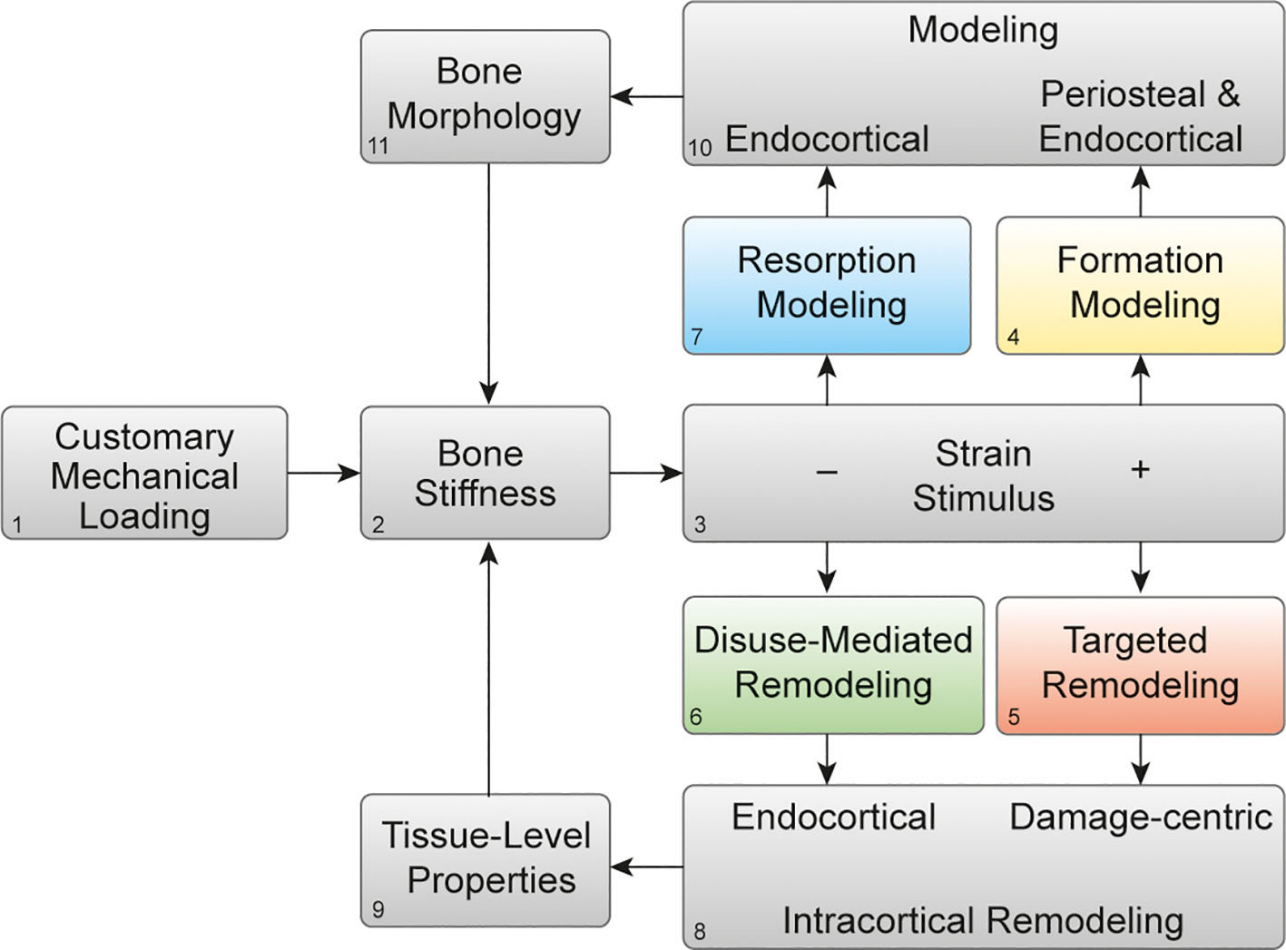
Mechanoadaptation of bone: Theoretical framework of bone functional adaptation at the diaphysis. Mechanical loading (1) on a bone of a given stiffness (2) will produce a strain stimulus (3), which, if greater than customary, may elicit bone formation modeling (4) or targeted remodeling (5). In the case of disuse and a lower than customary strain stimulus, disuse-mediated remodeling (6) and resorption modeling (7) may occur. Remodeling near damage or the endocortical surface (8) primarily alters tissue-level mechanical properties (9), whereas modeling on the periosteal or endocortical surfaces (10) alters the bone morphology (11). Bone morphology and tissue-level properties collectively determine whole-bone stiffness (2), which then influences the strain response to subsequent bouts of mechanical loading. Reprinted with permission from Hughes et al., Exercise and Sport Sciences Reviews, 2020 (2).

Targeted remodeling is damage-centric and can occur following osteocyte expression of genes associated with osteoclastogenesis within 100 and 300 microns of linear microdamage (11) in cortical and trabecular bone. Disuse-mediated remodeling occurs near the endocortical surface (**Figure 2.8**), and both types of remodeling will influence the tissue-level properties of the bone (**Figure 2.9**). Bone modeling which occurs on the periosteal and endocortical surfaces of the long bone diaphysis in formation modeling and on the endocortical surface in resorption modeling (**Figure 2.10**), and by altering the size, shape, and cortical thickness of the bone, will ultimately influence the morphology (**Figure 2.11**). In turn, changes in the bone morphology and tissue-level properties will together alter bone stiffness in a negative feedback fashion (**Figure 2.2**). We propose that this simple negative feedback model can help decipher the intricate interactions between mechanical and hormonal regulation of bone stiffness, as we discuss below.

### 2.2 Hormonal integration with mechanical regulation

There are three tissue-level modes of increasing bone stiffness in response to hormonal stimulation. The first mode is through formation modeling on trabecular, endocortical, or periosteal surfaces which can increase trabecular and cortical thickness (12). A second mode for increasing bone stiffness is by enhancing osteoblastic activity within existing remodeling units (13), and a third mode is by preventing bone resorption altogether (14), whether by suppressing the activation of new remodeling cycles or preventing resorption modeling. These mechanisms, whether through bone anabolism or prevention of resorption, by increasing bone stiffness, will alter the customary strain stimulus and initiate bone functional adaptation. In each of the examples below, we begin with well-established observations of hormone-mediated increases in bone stiffness. We then review adaptive phenomena that occur after hormone-mediated increases in stiffness, whose foundations in mechanical regulation of bone are not always recognized. We conclude each example by highlighting the potential of exercise to offset bone resorption or promote bone formation that can accompany bone functional adaptation after hormone-induced increases in bone stiffness.

### 2.3 Hormonal stimulation of increased bone stiffness in adolescent boys and girls

During growth, long bones increase in length by endochondral ossification, a process that is largely driven by pulsatile secretion of growth hormone (GH) and other growth factors (4, 15). Endochondral ossification concludes by estrogen-induced closure of the growth plates in girls two years earlier than in boys, who experience a rise in estrogen via the aromatase pathways (16). Recently, animal studies have demonstrated that sex-based differences in GH secretion, favoring males, may have epigenetic foundations (17–20). The longitudinal pubertal growth in bone may provide some of the mechanical stimulus that drives radial growth through deposition on the subperiosteal surface, and radial growth also has roots in hormonal stimulation (4, 21). During puberty, periosteal apposition is particularly accelerated in boys relative to girls (22), and the resultant structural advantage of greater bone size is attributed to exposure to greater testosterone, growth hormone, and insulin-like growth factor-1 (IGF-1) concentrations during puberty in boys than in girls (23, 24). However, testosterone could also indirectly increase bone size through stimulating increases in muscle mass leading to greater mechanical loading (25). Bone stiffness increases disproportionately relative to the amount of new bone mass that is formed when formation modeling occurs on the periosteal surface of the diaphysis of long bones (26). This is because periosteal apposition occurs on the surface furthest from the neutral axis in bending and therefore increases the cross-sectional moment of inertia (26, 27). Therefore, formation modeling that leads to periosteal apposition in an accelerated manner in boys during puberty should disproportionately increase bone stiffness in boys relative to girls. There are mechanical advantages that accompany the increase in stiffness, with boys attaining 28% to 63% greater strength of the appendicular long bones across longitudinal growth, compared to biologically age-matched girls (28).

**Figure 3:**
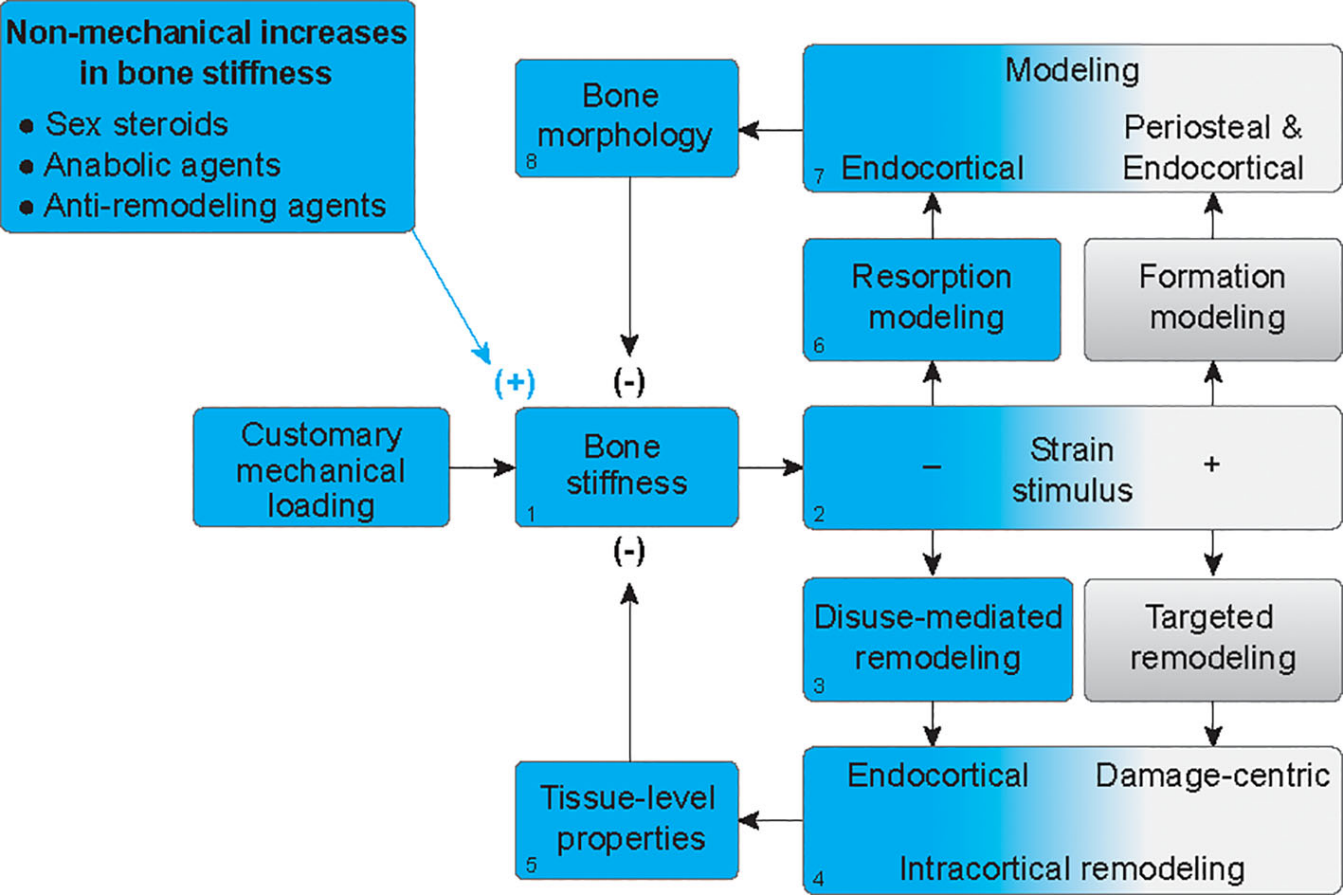
Mechanoadaptation of bone after hormone-mediated increases in bone stiffness. In the absence of proportionally greater increases in customary mechanical loading, when bone stiffness is increased by hormonal stimuli (1), strain stimuli will fall below customary ranges (blue gradient) (2), thus eliciting disuse-mediated remodeling (3), predominantly near the endocortical surface (4). Intracortical remodeling with a negative bone balance, will increase tissue porosity (5), leading to decreased bone stiffness (1). Decreased strain stimuli will simultaneously elicit resorption modeling (6), also predominantly at the endocortical surface (7). This resorption, independent of formation, will expand the marrow cavity (8). Together, increased tissue porosity and endocortical expansion will decrease bone stiffness to homeostatic levels (1).

Although boys have a mechanical advantage in terms of overall bone strength, they also have 28% to 80% more porous cortices than girls (28). The physiologic foundations for the greater increases in cortical porosity throughout growth in boys, relative to girls, is unknown but has been attributed to the need for bones to not only remain stiff and strong but also minimally massive (29). Indeed, bone tissue is approximately twice as dense as other body tissues and therefore requires more energy to move during locomotion (3). We propose a novel way to at least partially explain the observations that men not only have wider bones but also more porous bones on average, compared to women. Specifically, we offer that increased porosity could be a compensatory mechanism that follows periosteal apposition (**Figure 3**). When forces from habitual mechanical loading are applied to a bone that has increased stiffness due to hormone-driven periosteal apposition (**Figure 3.1**), the stiffer structure will deform less, and strain stimuli will fall below customary ranges (**Figure 3.2**). This will elicit disuse-mediated remodeling (**Figure 3.3**), predominantly near the endocortical surface (**Figure 3.4**). Intracortical remodeling with a negative bone balance will increase tissue porosity (**Figure 3.5**), leading to decreased bone stiffness (**Figure 3.1**). Decreased strain stimuli will simultaneously elicit resorption modeling (**Figure 3.6**), also predominantly at the endocortical surface (**Figure 3.7**). This resorption, independent of formation, will expand the marrow cavity (**Figure 3.8**). Together, increased tissue porosity and endocortical expansion will decrease bone stiffness (**Figure 3.2**) and subsequent strain stimuli will return to equilibrium, as is the hallmark of negative feedback loops. Thus, bone loss after periosteal apposition during growth may occur at least partially because of a perceived disuse that accompanies increases in bone stiffness without parallel increases in mechanical loading.

While boys experience endocortical expansion during adolescence, they do so to a greater degree than girls (22), leading to narrower medullary cavities in adult women, on average, than in men. Not only are girls not exposed to the concentrations of androgens and growth hormones that lead to accelerated periosteal expansion in boys, but girls also experience greater increases in concentrations of estrogen (30). The anti-resorptive effects of estrogen are well-established (30), and the physiologic model in **Figure 3** would predict that inhibition of bone resorption by estrogen, combined with less periosteal expansion than that observed in boys, would preserve bone stiffness and maintain customary ranges of strain stimuli during mechanical loading. Ultimately, this would lead to a lack of stimulus for endocortical expansion in girls compared to boys. Collectively, hormonal and mechanical co-regulation of bone stiffness can provide physiological context for sex-based differences in the adult skeleton, with men having wider, more porous bones and expanded medullary cavities than women (22, 31).

### 2.4 Exercise to prevent perceived disuse and increase mechanosensitivity in adolescence

**Figure 4:**
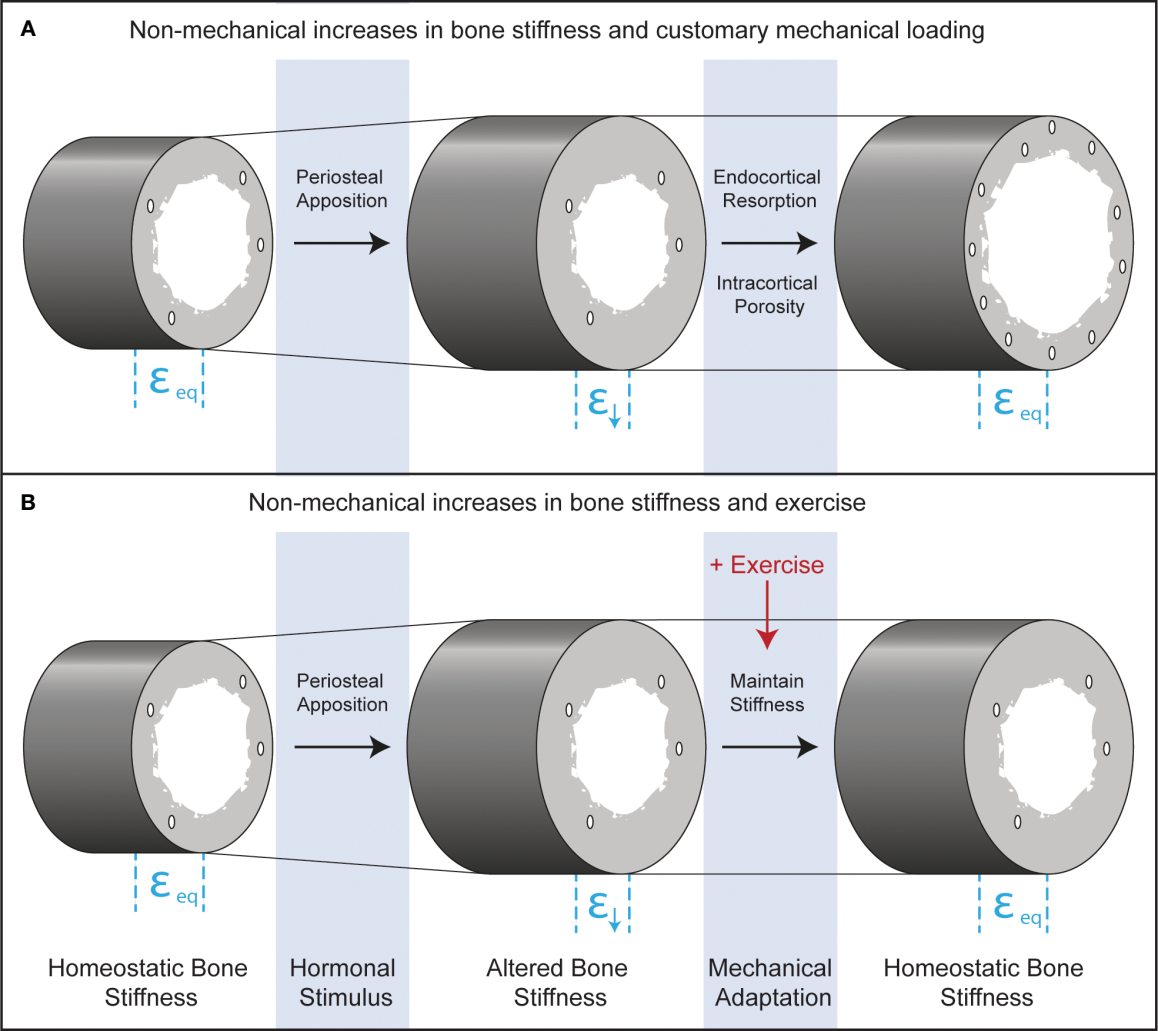
Longitudinal bone cross-sections depicting endocrine and mechanical co-regulation of bone stiffness after androgen- and TPTD-driven periosteal apposition. Before hormonal stimulation, strains during customary mechanical loading will be at equilibrium (ε_eq_). Periosteal apposition increases the diameter of the bone and therefore the stiffness. Increased stiffness leads to declines in strain during customary mechanical loading (ε↓), which could lead to functional adaptation of bone through increased resorption at the endocortical surface or through intracortical remodeling that increases porosity. These adaptive responses will bring strains to homeostatic levels. Exercise may inhibit adaptive bone resorption, leading to maximal gains in bone formation from the initiating hormonal stimulus.

The perceived disuse that may accompany increases in bone stiffness from periosteal apposition in boys is counterintuitive because it does not include classic disuse conditions such as bed rest, limb immobilization, or microgravity and occurs in young healthy adolescents, absent of disease. Nonetheless, both traditional and perceived disuse occur when strain stimuli fall below customary levels, stimulating bone resorption. The practical implication of recognizing adaptive resorption is that increasing the habitual mechanical forces on bone through exercise could prevent it (**Figure 4**). If bone stiffness is increased from non-mechanical stimuli such as hormonally driven periosteal apposition, but there is a simultaneous increase in the mechanical loads placed on bone with exercise, this may maintain strain stimuli within a customary range and prevent disuse-mediated resorption.

For girls, exercise could capitalize on the increased mechanosensitivity of bone that accompanies estrogen exposure (32–34) and ultimately help attain a stronger adult skeleton. Promoting exercise during adolescent growth may hold other advantages for both sexes beyond preventing perceived disuse in boys and capitalizing on increased mechanosensitivity in girls. During puberty, growth in width lags behind growth in length, potentially creating a vulnerable window for increased risk of fracture in the long bones of the extremities (35). It is conceivable, though not yet experimentally demonstrated that exercise during this vulnerable period could stimulate more rapid periosteal expansion and help offset increased fracture risk. Finally, adolescence may be an opportune time to participate in exercise to stimulate adaptive bone formation during growth, because mechanosensitive osteoblasts are active along much of the bone surface due to longitudinal and appositional growth than during other times in life. There is a large body of literature supporting these concepts that exercise when young holds promise for building strong bones and offsetting the risk of osteoporosis and related fractures in adulthood for men and women (36–39). Even though men have greater bone strength and lower risk of osteoporotic fractures than women, osteoporotic fractures are still common in men who account for 30% of hip fractures (40). Therefore, prevention of disuse-mediated bone resorption during growth in boys and capitalizing on increased mechanosensitivity in girls with exercise may be an important strategy to decrease fracture risk later in life in both men and women.

### 2.5 Increased bone stiffness from intermittent parathyroid hormone and other osteoporosis treatments

Daily injections of PTH (1-84) or its analog, TPTD [PTH (1-34)], are osteoanabolic. The mechanisms whereby PTH or its analogs influence bone cells are numerous (13, 41). TPTD was the first anabolic drug available for treatment of osteoporosis, and histomorphometric evaluation of transiliac crest biopsies revealed that TPTD stimulates bone formation on all four primary bone surfaces (12). A classic study provided evidence that TPTD may initiate bone formation from bone lining cells, which are flattened osteoblastic cells aligned along quiescent surfaces (42). This theory was confirmed in a lineage tracing study reporting that three days of PTH administration resulted in *in vivo* conversion of normally flattened bone lining cells into cuboidal, mature osteoblasts (43). Thus, early anabolic action of intermittent PTH and TPTD is likely modeling-based. In cortical bone, formation modeling with TPTD can occur on both the endocortical and periosteal surfaces (12, 44). Biopsy studies also report increases in bone formation within existing remodeling units (12, 45, 46) and even reveal a novel remodeling phenomenon by which TPTD stimulates bone formation within the remodeling unit in such an accelerated manner that “overflow remodeling” can occur. In overflow remodeling, bone formation not only fills the resorption lacunae previously hollowed out by osteoclasts but also pours out over the lacunar boundary and onto previously quiescent surfaces (12). Collectively, these three modes of bone formation increase bone stiffness (47) with TPTD administration.

While bone formation is the hallmark of osteoanabolic drugs, much less is known about the mechanistic foundations for a delayed resorption after initiation of treatment with an intermittent PTH analog. Biopsy studies and serum biochemical markers of bone formation reveal that bone formation activity increases rapidly once TPTD is initiated, peaks within six to 12 months, and remains elevated above baseline values for at least 3 years of treatment (12, 45, 46, 48). After the first month of TPTD treatment, biochemical markers of bone resorption also begin to increase, and like markers of bone formation, also peak at 6-12 months, followed by a steady decline, but remaining elevated over baseline (49, 50).

The physiological foundations for increasing bone resorption after TPTD administration are not fully delineated. Coupling mechanisms between osteoblasts and osteoclasts such as secretion of RANKL, which promotes differentiation of osteoclasts (51), may partially explain increases in bone resorption. Co-regulation of bone stiffness by hormonal and mechanical stimuli can also account for these observations. When TPTD increases bone formation, bone stiffness is also increased (**Figure 3.1**). Without a simultaneous increase in habitual mechanical loading, strain stimuli will fall below customary levels (**Figure 3.2**), thus eliciting disuse-mediated bone remodeling (**Figure 3.3**), particularly at the endocortical surface (**Figure 3.4**). If TPTD continues to increase bone stiffness, independent of increases in mechanical loading or changes to sex steroids, bone remodeling should continue. When complete, disuse-mediated remodeling results in a negative bone balance and therefore increased porosity (**Figure 3.5**), leading to decreased bone stiffness and equilibrium (**Figure 3.2**). Resorption modeling, the other physiologic response to lower than customary strain stimuli, is not observed with TPTD, likely because endocortical lining cells that have converted to active osteoblasts may be actively forming bone along the surface, and during disuse, osteoclasts appear to avoid regions of ongoing osteoblast activity (52). Thus, we propose that increased but delayed bone resorption after TPTD treatment is partially due to a disuse-mediated increase in bone remodeling.

Besides anabolic drugs, other pharmacologic agents can increase bone stiffness. These include anti-resorptive drugs such as bisphosphonates, which are incorporated into the mineralized matrix and promote apoptosis of mature osteoclasts when released by the acid environment underneath active osteoclasts (53). After long-term treatment with bisphosphonates, brief periods of cessation can be introduced to prevent rare negative consequences of long-term remodeling suppression (54). Another anti-resorptive drug is denosumab which is not accumulated into the bone matrix and inhibits osteoclast differentiation, activity, and survival of osteoclasts (53). Inhibiting bone resorption by these drugs results in increased bone density, stiffness, strength, and ultimately, prevention of osteoporotic fractures (55). Discontinuation of drugs that increase bone stiffness can lead to increased bone resorption, which can reverse the drug’s effect on bone and fracture risk (54, 56–58). We propose this increased activation of bone resorption is in part due to adaptive bone resorption. If anabolic or anti-resorptive drugs increase bone stiffness (**Figure 3.1**), absent of proportional increases in customary mechanical loading, strain stimuli will be decreased (**Figure 3.2**), and bone functional adaption in the mode of disuse-mediated bone remodeling (**Figure 3.3**) or resorption modeling (**Figure 3.6**) will ensue. These processes would lead to increased porosity (**Figure 3.5**) and endocortical resorption (**Figure 3.7**), respectively, that would decrease bone stiffness (**Figure 3.1**) until homeostasis is achieved (**Figure 3.2**).

One strategy that has been effective for counteracting bone resorption after drug discontinuation is sequencing anabolic followed by anti-resorptive drugs (59–61). By providing drugs that inhibit resorption, loss from perceived disuse following non-mechanical increases in stiffness can be prevented. Although studies have demonstrated a benefit of sequencing anabolic therapy first, followed by anti-resorptive therapy, an unfortunate extension of our model would be that if the anti-resorptive therapy is eventually ceased, functional adaptation of bone would result in disuse-mediated bone loss. Relatively new therapies that both induce bone anabolism and inhibit bone remodeling, like the anti-sclerostin antibody, romosozumab, may help prevent bone resorption that accompanies cessation of drug therapy (62).

### 2.6 Exercise to prevent post-osteoanabolic therapy resorption

Increasing customary mechanical loading with exercise is a non-pharmacologic means for blunting disuse-mediated bone resorption after increases in bone stiffness. Increases in bone-loading exercise also holds promise as a companion to osteoporosis drugs for offsetting disuse-mediated bone loss. Prevention of disuse-mediate bone resorption by exercise has been demonstrated in classic disuse scenarios such as spinal cord injury, space flight, and bedrest (63–65). Our model would predict that a combination of exercise and anti-resorptive drugs would synergistically attenuate bone loss during disuse, as has been demonstrated in animal models with hindlimb unloading (66) and in astronauts due to microgravity (67). Combining exercise with osteoanabolic therapy may be successful in offsetting resorption that accompanies drugs that increase bone stiffness. In a rodent model of type 2 diabetes, TPTD and exercise separately increased lumbar spine BMD, but only the two combined improved trabecular and cortical bone microarchitecture and breaking strength (68). Similarly, combined TPTD and exercise improved bone structure and strength of cortical bone at the femoral diaphysis in ovariectomized and tail-suspended rats (69). In postmenopausal women with osteoporosis, TPTD and whole-body vibration exercise had synergistic effects in increasing lumbar spine BMD, compared to TPTD alone (70).

Exercise may also attenuate the rapid increase in bone resorption with cessation of osteoporosis therapy. TPTD administered for 12 weeks partially prevented bone loss in ovariectomized rats. After cessation of TPTD, eight weeks of treadmill exercise maintained the TPTD-stimulated bone anabolism, compared to non-exercise controls whose bone mass returned to pre-TPTD levels in eight weeks (71). Whether exercise can combat bone loss after cessation of osteoporosis therapies in humans remains to be determined. Studies of exercise in women and men with osteoporosis who cease osteoporotic drug therapies are needed to determine if exercise can indeed prevent bone resorption.

## 3 Summary

In this review, we discussed the concept that when bone stiffness is increased due to hormonal stimuli, without simultaneous increases in mechanical loading, disuse-mediated resorption can occur and offset some of the mechanical advantages of hormone-induced gains in bone tissue. Recognition of these integrated physiological processes provides a framework for interpreting successive adaptions. Recognizing these interactions also highlights the benefits of exercise for preventing disuse-mediated bone resorption that can follow increases in bone stiffness during adolescence, osteoanabolic drug therapy, and following cessation of osteoporosis treatments, thus providing further support for the important role for exercise in offsetting skeletal fragility throughout life.

## Author contributions

JH conceived of the manuscript. CC designed the figures. SP revised the manuscript. All authors contributed to the article and approved the submitted version.
